# Linking Biochemical and Structural States of SERCA: Achievements, Challenges, and New Opportunities

**DOI:** 10.3390/ijms21114146

**Published:** 2020-06-10

**Authors:** Rodrigo Aguayo-Ortiz, L. Michel Espinoza-Fonseca

**Affiliations:** Center for Arrhythmia Research, Department of Internal Medicine, Division of Cardiovascular Medicine, University of Michigan, Ann Arbor, MI 48109, USA; raguayoo@umich.edu

**Keywords:** SERCA, calcium, X-ray crystallography, P-type ATPase, inhibition, regulation, biophysical methods, molecular simulation

## Abstract

Sarcoendoplasmic reticulum calcium ATPase (SERCA), a member of the P-type ATPase family of ion and lipid pumps, is responsible for the active transport of Ca^2+^ from the cytoplasm into the sarcoplasmic reticulum lumen of muscle cells, into the endoplasmic reticulum (ER) of non-muscle cells. X-ray crystallography has proven to be an invaluable tool in understanding the structural changes of SERCA, and more than 70 SERCA crystal structures representing major biochemical states (defined by bound ligand) have been deposited in the Protein Data Bank. Consequently, SERCA is one of the best characterized components of the calcium transport machinery in the cell. Emerging approaches in the field, including spectroscopy and molecular simulation, now help integrate and interpret this rich structural information to understand the conformational transitions of SERCA that occur during activation, inhibition, and regulation. In this review, we provide an overview of the crystal structures of SERCA, focusing on identifying metrics that facilitate structure-based categorization of major steps along the catalytic cycle. We examine the integration of crystallographic data with different biophysical approaches and computational methods to link biochemical and structural states of SERCA that are populated in the cell. Finally, we discuss the challenges and new opportunities in the field, including structural elucidation of functionally important and novel regulatory complexes of SERCA, understanding the structural basis of functional divergence among homologous SERCA regulators, and bridging the gap between basic and translational research directed toward therapeutic modulation of SERCA.

## 1. Introduction

Sarcoendoplasmic reticulum calcium ATPase (SERCA) is a transmembrane pump critical for Ca^2+^ transport from the cytosol into the sarcoplasmic reticulum (SR) lumen in skeletal and cardiac muscle cells, and into the endoplasmic reticulum (ER) of non-muscle cells [1]. The protein structure is highly conserved, exhibiting variations only in the extension of the C-terminal region across isoforms and splice variants. Nine splice variants of SERCA (SERCA1a-b, SERCA2a-d, SERCA3a-c) encoded by three different genes (*ATP2A1-3*) have been identified in humans [2]. SERCA1a (994 aa) and SERCA1b (1011 aa) isoforms are normally expressed in adult and fetus skeletal muscle, respectively [3]. The cardiac muscle mainly expresses the SERCA2a (997 aa) variant, whereas the SERCA2b (1042 aa) isoform is located in nerve cells [4,5]. The C-terminal region of SERCA2b has been associated to alterations in the Ca^2+^ interaction and the transition between low-Ca^2+^ affinity states [6]. The distribution and function of SERCA3 (999–1052 aa) splice variants have not been fully understood [7], although studies have shown that Ca^2+^ affinity is inherently lower for SERCA3 than for other SERCA isoforms [8]. Despite their remarkable similarities among SERCA isoforms, there are differences in calcium affinity and enzyme kinetics [3,8,9,10,11].

**Figure 1:**
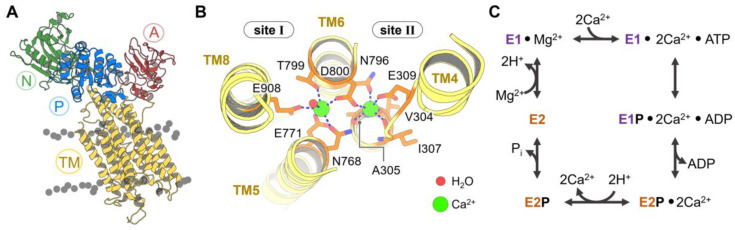
(**A**) Three-dimensional structure of the cytosolic N (green), A (red), and P (blue) domains and transmembrane helices (TM, yellow) of sarcoendoplasmic reticulum calcium ATPase (SERCA). (**B**) Close-up view of calcium binding sites I and II of SERCA showing two Ca^2+^ ions (green) and the interactions stabilizing their position in these sites. (**C**) Schematic representation of the Post-Albers pumping cycle of SERCA.

SERCA uses the energy produced by ATP hydrolysis to transport two Ca^2+^ ions from the cytosol into the SR lumen in exchange for two protons exported from the SR lumen to the cytosol (Figure 1) [12,13]. During the catalytic cycle of the pump, SERCA populates two major structural states: A high-Ca^2+^ affinity state (E1), and a low-Ca^2+^ affinity state (E2). Each catalytic cycle begins with the formation of the E1 state that features transmembrane (TM) transport sites that are exposed to the cytosol; this E1 state binds two Ca^2+^ ions from the cytosol TM into the transport sites and one molecule of ATP in the nucleotide-binding domain. The ATP-bound E1-2Ca^2+^ complex induces a structural arrangement of the pump that facilitates the coupling of ATP hydrolysis and Ca^2+^ transport; ATP hydrolysis then favors the formation of a phosphorylated SERCA intermediate, E1~P-ADP-2Ca^2+^ [14]. This step in the catalytic cycle induces a structural transition toward a phosphorylated E2P-2Ca^2+^ state that has low affinity for Ca^2+^, thus inducing dissociation of two Ca^2+^ ions from SERCA and their release into the SR/ER lumen. Upon Ca^2+^ dissociation from SERCA, two luminal protons diffuse into the transport sites to stabilize a phosphorylated, low-Ca^2+^ affinity E2-P state [15,16,17,18]. This step is followed by SERCA dephosphorylation [19,20] required to populate the apo E2 state of the pump [14,21,22]. Finally, two protons are spontaneously released from the transport sites to the cytosol [15,23,24], thus accelerating the E2-to-E1 structural transition that is required to populate the E1 state for the next Ca^2+^-pumping cycle [21,22].

SERCA regulatory partners have been identified for isoforms 1a, 2a, and 2b, and most of these are inhibitory membrane proteins that mediate tissue-specific regulation of the pump. SERCA is primarily regulated by two analogous transmembrane proteins: The 52-residue phospholamban (PLB), which is primarily expressed in ventricles and slow-twitch skeletal muscle; and the 31-residue sarcolipin (SLN), which is predominantly expressed in fast-twitch skeletal muscle and atria [25,26]. PLB and SLN share substantial transmembrane domain homology, and both bind to the same canonical TM2/TM6/TM9 groove of SERCA to form 1:1 heterodimeric complexes [27,28,29] with distinct functional outcomes [30,31,32,33]. PLB and SLN inhibit SERCA by decreasing Ca^2+^ affinity, while SLN also decreases *V_max_* (activity at high Ca^2+^). Nonetheless, the effect of SLN on the *V_max_* of SERCA remains controversial because independent studies have proposed that this protein either stimulates or has no effect on the *V_max_* of the pump [34,35]. These differences confer PLB and SLN with unique functional roles in their mechanisms for SERCA regulation of muscle contractility. For instance, it is known that SLN, but not PLB, contributes to non-shivering thermogenesis in skeletal muscle [36] by inducing uncoupling of Ca^2+^ transport from ATP hydrolysis by SERCA, thereby stimulating unproductive ATP hydrolysis and heat production [37,38]. To illustrate the functional differences between PLB and SLN, studies have shown that ablation of SLN in mice results in an obese phenotype when fed a high-fat diet, whereas those over-expressing SLN are protected from diet-induced obesity [39]. Conversely, PLB-null mice are not predisposed to diet-induced obesity or glucose intolerance when fed a high-fat diet [40], thus showing that only SLN-induced uncoupling of SERCA enhances energy expenditure [41].

A major challenge in the field is to understand in atomic-level detail the mechanisms for SERCA activation and regulation in terms of the interactions and structural changes of the underlying proteins. X-ray crystallographic studies have helped overcome this challenge, and have provided unique insights into the interactions, structural changes, and intermediates that SERCA populates as it progresses through activation and regulation during the transport cycle. The rich structural information from these studies and the advances in spectroscopy and molecular simulation now provide unique mechanistic insight into SERCA function and regulation in unprecedented spatial and temporal resolution. In this review, we summarize the advances and achievements toward linking biochemical and structural states of SERCA, and we discuss the challenges and new opportunities in the field, emphasizing its importance in regulation and its potential use as a therapeutic target.

## 2. Crystal Structures of SERCA: General Considerations

To date, 76 crystal structures of SERCA have been deposited in the Protein Data Bank (PDB): 72 of SERCA1a, two of SERCA2a and two of SERCA2b. Table A1 (see Appendix A) shows the biochemical state, code, resolution, and bound ligands found in crystal structures reported in the PDB. Except for two crystal structures, *1kju* and *3j7t*, three-dimensional structures were obtained using X-ray crystallography at resolutions between 2.1 and 4.0 Å. The remaining structures were obtained using electron microscopy (EM, *1kju*) [42] and electron crystallography (EC, *3j7t*) [43] methods. The structure of SERCA is incomplete in two reported crystal structures: *1kju*, where the resolution was too low (6 Å [42]), and *4nab,* where the loss-of-function mutation E309Q interfered with complete mapping of the full-length structure of the pump [44].

**Figure A1:**
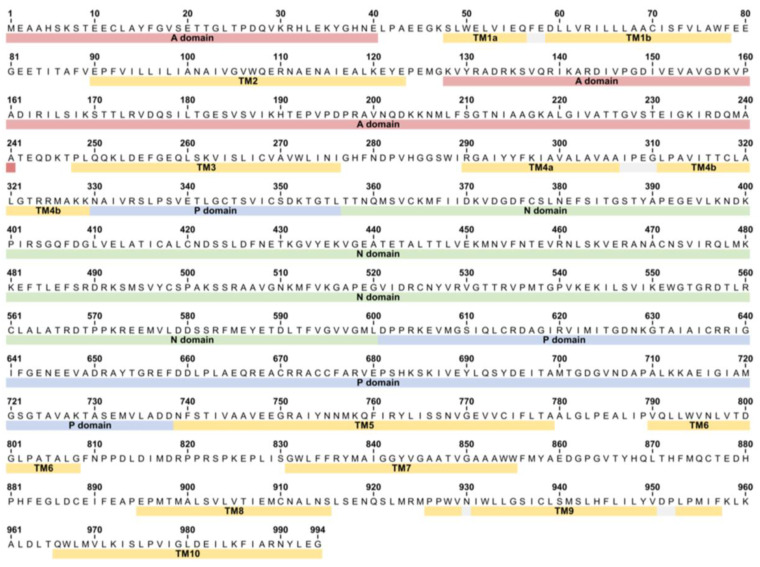
Sequence of SERCA1a from rabbit (*Oryctolagus cuniculus)* showing the location of all major domains.

X-ray crystallography studies have shown that SERCA is characterized by a TM domain and a cytosolic headpiece (Figure 1A and Figure A1 of Appendix B). The TM domain is composed of 10 transmembrane helices (TM1-TM10) that contain the negatively charged Ca^2+^ transport sites I and II; these transport sites are located within a pocket delineated by TM helices TM4, TM5, TM6, and TM8 (Figure 1B) [45]. The cytosolic headpiece houses the catalytic elements required for the coupling of ATP hydrolysis with subsequent Ca^2+^ transport [14,46,47,48] and is formed by three functional domains: Nucleotide-binding (N), phosphorylation (P), and actuator (A) domains. During catalytic processing, the N domain is responsible for binding to the ATP nucleotide, bringing it closer to the phosphorylation site in the P domain at position D351. The A domain serves as the transduction element that couples ATP hydrolysis with active Ca^2+^ transport in the catalytic cycle of the pump [49].

**Figure 2:**
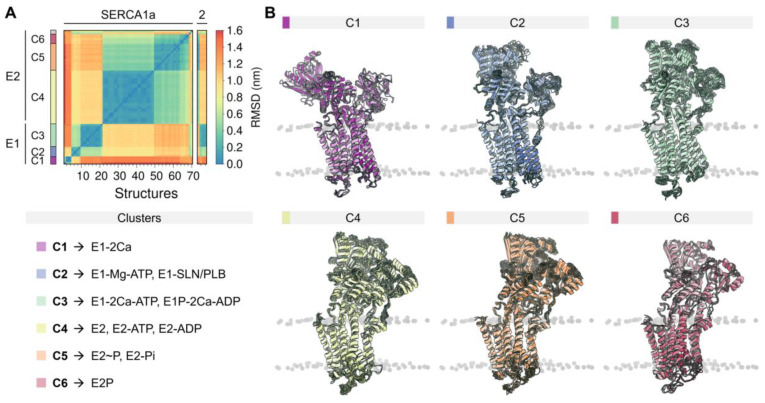
(**A**) Backbone root-mean-square deviation (RMSD) matrix of SERCA1a and SERCA2a/b structures and state information of the six clusters found. (**B**) Depiction of the superimposed SERCA structures comprising the six major clusters. SERCA1a structures *1kju* and *4nab* were excluded from the analysis because they were obtained at low resolution, or because they were missing large regions/domains in the reported structure. Short loops and missing side chains were built to perform the backbone alignment (see methods). Clustering analysis was performed using a cut-off RMSD value of 0.23 nm.

**Figure A2:**
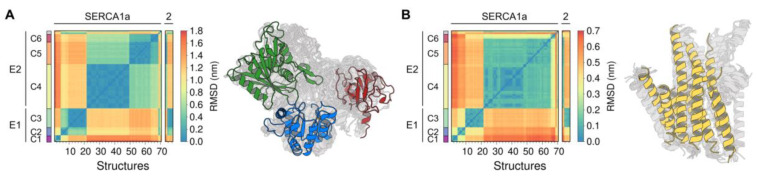
Root-mean-square deviation (RMSD) matrix calculated for the (**A**) Cytosolic domains and (**B**) TM domain of SERCA1a (70 structures), SERCA2a (2 structures), and SERCA2b (2 structures). RMSD-based clustering shows the presence of six major structural clusters, C1 through C6.

The rich structural information now allows us to understand the conformational distribution and similarities of the different structures deposited in the PDB. Root-mean-square deviation (RMSD)-based clustering analysis of 74 crystal structures of the pump shows that SERCA populates 6 major clusters that summarize the coupling between structural and biochemical states of the pump (Figure 2). These clusters recapitulate the conformational changes of the cytosolic domains during the catalytic cycle of the pump and can help identify more generally the structure of SERCA’s E1 and E2 states (see Figure A2 of Appendix B). Table A1 reports the conformational cluster of each SERCA structure and the biochemical state according to the Post-Albers scheme. This analysis shows that *Cluster 1* (*C1*), represented by the crystal structure of Ca^2+^-bound SERCA (E1-2Ca), displays a unique structural arrangement in which all three cytosolic domains are pulled apart to populate an “off” state that disfavors ATPase activity. The high structural similarity between E1-ATP-Mg and E1-SLN/PLB states (*Cluster 2, C2*) suggests that Mg^2+^/ATP and PLB/SLN promote a conformational change that resembles *C1*, albeit with a substantially more closed structure of the headpiece. This cluster is characterized by a structure where the cytosolic N domain is close to the P domain; however, the N and A domains remain apart in cluster *C2*, thus also rendering SERCA unable to couple ATP hydrolysis and subsequent Ca^2+^ transport across the SR. Conversely, E1 state structures in the presence of bound nucleotide and Ca^2+^ ions (*Cluster 3, C3*) exhibit a compact headpiece geometry that brings together the catalytic elements of the headpiece required for activation of ATPase activity. The structures of the E2 state differ substantially from the E1 state predominantly by the changes in rotation and displacement of the A domain and the structural rearrangements of the TM domain. Specifically, *Cluster 4* (*C4*) retains a closed conformation of the cytosolic domains that resemble that of cluster *C3* but with some differences in the arrangement of the A domain. Unlike the E1 state, where ATP/ADP-bound and unbound forms are distinguishable between SERCA structures (*C1* to *C3*), E2 state conformers grouped in *C4* resembles E2-ATP and E2-ADP state conformations (RMSD < 0.2 nm). The phosphorylated E2 intermediate states of the pump (i.e., E2P- and E2-Pi-like), grouped in *Cluster 5* (*C5*), exhibit clear differences in the RMSD (0.43 ± 0.03 nm) compared to the unphosphorylated E2 state (cluster *C4*). Finally, structures representing in the E2/E2P states with bound Mg^2+^ at site II (*Cluster 6*, *C6*) are characterized by partial separation of the cytosolic domains, suggesting that this structural state represents a conformation of the pump that is populated at the beginning of the E2-to-E1 transition. It is worth noting that in our general analysis, crystal structures *5zmw* and *5ncq* could not be unequivocally grouped into any of the clusters described here. The reasons for this observation are varied and complex but are likely related to the unique structural changes induced by a point mutation targeting the transport site II (i.e., E309Q in *5zmw* [50]) or the unique structural arrangement induced by small-molecule inhibition of the pump (i.e., tetrahydrocarbazole in *5ncq*, see Section 4) [51].

## 3. Dissecting Structural Changes in the Catalytic Cycle of SERCA

During active Ca^2+^ transport, SERCA undergoes large structural changes in its functional domains as it harnesses the energy produced by hydrolysis of ATP. These changes in the structure and orientation of the headpiece, as well as in the topology of the TM domain, are key to enable the exquisite coupling between ATPase activity with Ca^2+^ transport into the SR/ER. In this section, we present a general analysis of the rich crystallographic data to dissect the structural changes that occur along the catalytic cycle of the pump. The result is a vivid atomic-resolution visualization of the dynamic nature of SERCA’s complex Ca^2+^ transport machinery.

### 3.1. The Cytosolic Headpiece of SERCA

**Figure 3:**
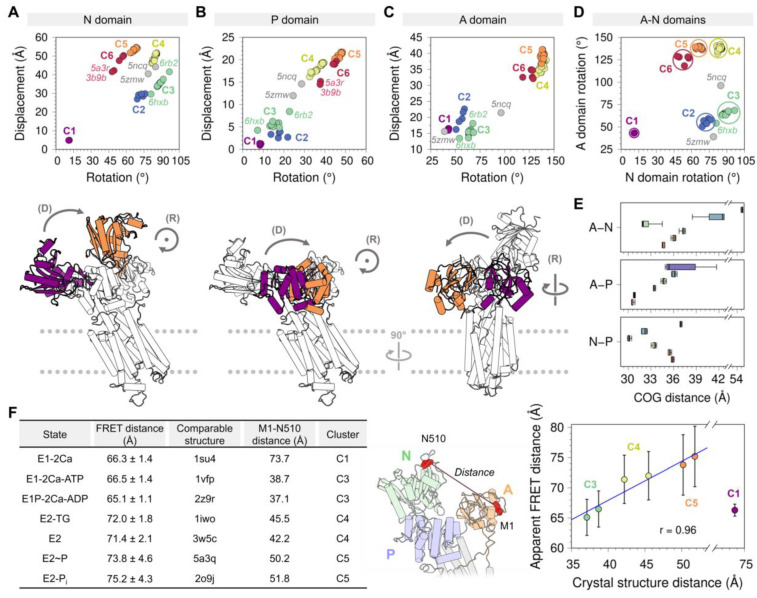
Displacement distances and rotation angles of the (**A**) N domain, (**B**) P domain, and (**C**) A domain recovered from the transformation matrix calculation for the 74 SERCA structures using *2c9m* (E1-2Ca state) as reference. The displacement (D) and rotation (R) movements of each of the domains are represented below each plot, coloring in purple and orange the initial and final positions. (**D**) Distribution of the six clusters based on the rotation of the A and P domains. (**E**) Distance between the center of geometry (COG) of A and N domains (top), A and P domains (middle), and N and P domains (bottom) for each cluster. (**F**) Fluorescence resonance energy transfer (FRET) and Met1-Asn510 distance comparison performed by Raguimova et al. [47] to analyze the population distribution of SERCA in response to dynamic changes in intracellular calcium. The figure in the middle represents the distance between the Met1 and Asn510 residues, the table and the graph contain the experimental and theoretical data generated by Raguimova et al. E1-2Ca state (*C1*) distance values were not considered for the linear regression. All plots are colored using the cluster analysis in Figure 2 as reference (*C1*, purple; *C2*, blue; *C3*, green; *C4*, yellow; *C5*, orange; *C6*, red).

Changes in the orientation of the cytosolic domain dictate the structural changes required for SERCA activation. To provide an unbiased analysis of these structural changes represented by crystal structures of the pump, we computed the transformation matrix of each cytosolic domain (N, P, and A) to measure displacement distances and rotation angles of these domains (Figure 3). In this subsection, we use this analysis to briefly discuss domain-specific structural changes observed in crystal structures of SERCA and their correlation with available spectroscopic and computational studies of the pump. Ultimately, these analyses provide help to establish relationships between motions and structural changes of the headpiece throughout the functional cycle of the pump, as well as better quantitative parameters that may be used to interpret structural data obtained in orthogonal studies linking the structure and function of SERCA in the cell.

#### 3.1.1. Structural Changes of the Nucleotide-Binding Domain

Analysis of the displacement distances and rotation angles of this domain correlates well with our initial clustering analysis showing the presence of a single-membered cluster *C1* (purple) (Figure 3A). The transition from clusters *C1* to *C2* and *C3* (blue and green, respectively) occurs primarily through a 55–79° clockwise rotation and a 20–30 Å displacement of the domain. This structural transition corresponds to the step in the cycle that brings together ATP to the phosphorylation site. It has also been proposed that this structural transition plays a role in nucleotide exchange during cycling of the pump, although this notion has been challenged by studies suggesting that the nucleotide is bound to SERCA throughout the entire reaction cycle, either in a catalytic or modulatory role [52,53]. The forward direction of the cycle toward the E2 state is accompanied by an increase in the displacement of the N domain (i.e., clusters *C4* to *C6*), and a counterclockwise rotation provides a means for repositioning the N domain to a structure similar to that in the E1 state (i.e., clusters *C1* through *C3*). Interestingly, we obtain a circle-shaped projection of the structures where different clusters can be linked to a certain step in the catalytic cycle if we exclude cluster *C1* from the rotation-displacement landscape of the N domain. In this arrangement of the structural landscape of the N domain, we find that configurations representing E1 states (clusters *C2* and *C3*) are clearly separated from the E2 states of the pump (clusters *C5* and *C6*), but cluster *C4* serves as a bridge between the two major group of configurations, thus providing a means to lower the energy barrier required for the N domain to adjust its position during the transitions between E1 and E2 state configurations (Figure 3A).

#### 3.1.2. Phosphorylation and Actuator Domains

Rotation angles and displacement distances of the P and A domains also capture the clustering profiles observed in the N domain, with well-defined populations for each state representing clusters *C1* through *C6*. Analysis of the crystal structures shows that the P domain adopts rotation angles less than 25° and displacement distances below 10 Å in E1 state structures, whereas greater rotation angles and displacement distances of this domain are found in crystal structures of the E2 state (Figure 3B). Crystal structures representing the E1 state are characterized by A domain rotation angles less than 90° and displacement distances less than 22 Å, while structures of the E2 state have rotation angles of the A domain larger than 100° and displacement distances larger than 26 Å (Figure 3C). An important observation here is that the structural landscape of the P and A domains is substantially different from that of the N domain: Unlike a circle-like landscape populated by the N domain, there is a clear linear relationship between displacement distances and rotation angles of the P and A domains during the E1-to-E2 transition. This suggests that P and A domain rotations occur in concert with their displacement during the transition between E1 and E2 states, while the N domain carries out a counterclockwise rotational change from *C2* to *C6* with negligible changes in displacement distances. Furthermore, the crystal structures of SERCA *5ncq* and *5zmw* represent intermediate states along with the rotation/displacement of the P domain, but not the N and A domains, during the E1 and E2 state transitions. These changes in domain displacement and rotation agree with the hypothesis that the crystal structure *5zmw* (E309Q mutant of SERCA1a) serves as a structural template describing an intermediate along with the E2-to-E1 transition of SERCA [50]. A similar hypothesis may be proposed using a recent crystal structure of SERCA1a mutant E340A (*6rb2* [54]). This mutation is known to induce phosphorylation overshoot caused by accumulation of dephosphoenzyme at steady state [55], so this crystal structure probably serves as a model to describe specific structural transitions occurring along the E1-to-E2 transition. Other structures of SERCA, such as *6hxb* [56], may also help identify the structure of SERCA intermediates along the catalytic cycle of the pump, but conclusions drawn from this structural information should be interpreted with caution, especially if the information suffers from low resolution.

#### 3.1.3. Correlation between Interdomain Movements and Functional Changes in the Catalytic Cycle of the Pump

**Figure A3:**
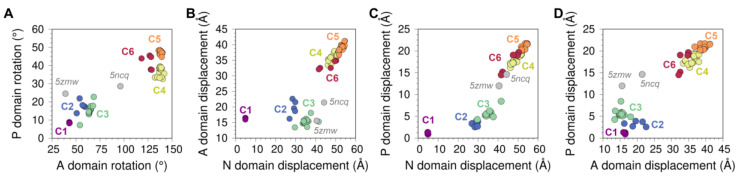
Rotation angle (R) calculated for (**A**) P-A domains and displacement distance (T) for (**B**) A-N, (**C**) P-N, and (**D**) P-A domains. A total of 74 SERCA1a structures were compared, using *2c9m* as reference.

The P and N domains are physically connected by two short linkers (residues L356-T357 and L600-D601), but the displacement and rotation of the P domain directly correlate with those of the A domain (see Figure A3 in Appendix B). This observation suggests that P and A domains’ movements are highly coupled during structural transitions between states during the catalytic cycle of the pump. Surprisingly, analysis of the crystal structures indicates that the relationships between rotation angle and displacement distances between these domains are insufficient to fully describe the complexity of the structural changes of SERCA during the structural transitions between E1 and E2 states. Nevertheless, this analysis revealed that rotation of the A and N domains provide a better separation of the structural states of SERCA (Figure 3D). This observation can be explained by the fact that rotation of the N and A domains are clearly distinguishable during the structural transitions between E1 and E2 states: The N and A domains undergo a clockwise and counterclockwise rotation during the E2-to-E1 transition, and rotations occur in the opposite direction (counterclockwise and clockwise for the N and A domains, respectively) during the E1-to-E2 transition [57]. This evidence supports the hypothesis that rigid-body motions of the N and A domains are a more accurate parameter that can be used for identification and classification of structural transitions of the headpiece during the catalytic cycle of SERCA.

Crystal structures corresponding to various states of the pump have shown that binding of Ca^2+^, nucleotides, small-molecule inhibitors, and regulatory proteins induces substantial structural changes in the cytosolic domains, transitioning between open and closed structural arrangements (Figure 3E). However, the extent of this structural change has been debated, and it has been suggested that SERCA occupies distinct structural states (i.e., open and closed) in a single biochemical state [58]. More recently, cell-based intramolecular fluorescence resonance energy transfer (FRET) experiments using a 2-color SERCA showed that except for the Ca^2+^-bound structure of SERCA, there is a linear relationship between interdomain N–A distances estimated with FRET, and those calculated directly from crystal structures of the pump (Figure 3F). More importantly, these studies showed that in the E2 state, SERCA populates predominantly open headpiece structures (i.e., clusters *C4* and *C5*), whereas the E1 state, represented here by the Ca^2+^-bound state (cluster *C1*), is characterized by a closed headpiece structure [47]. More recently, complementary time-resolved X-ray solution scattering (TR-XSS) and atomistic simulations were used to elucidate the structural dynamics of SERCA along the reaction cycle [59]. These studies revealed important features of the structural and functional dynamics of the pump, including: (1) The crystal structure of the open Ca^2+^-bound state (i.e., cluster *C1* here) is not representative of the structural ensemble of SERCA in this state, which populates a more compact structure in solution; and (2) SERCA populates a novel structure that corresponds to an ADP-sensitive Ca^2+^-bound E1P intermediate state of the pump. Complementary molecular dynamics simulations, mutagenesis studies, and FRET experiments have also helped understand the relationships between structural changes in the headpiece and SERCA activation, thus providing novel insights into the modular architecture of the cytosolic headpiece [46,47]. These studies are a prime example of a multidisciplinary approach for investigating real-time structural changes that SERCA undergoes in the native membrane while performing its function, and they offer a vivid picture of the structural mechanisms and transitions by providing snapshots of SERCA in action.

### 3.2. The Transmembrane Domain of SERCA 

**Figure 4:**
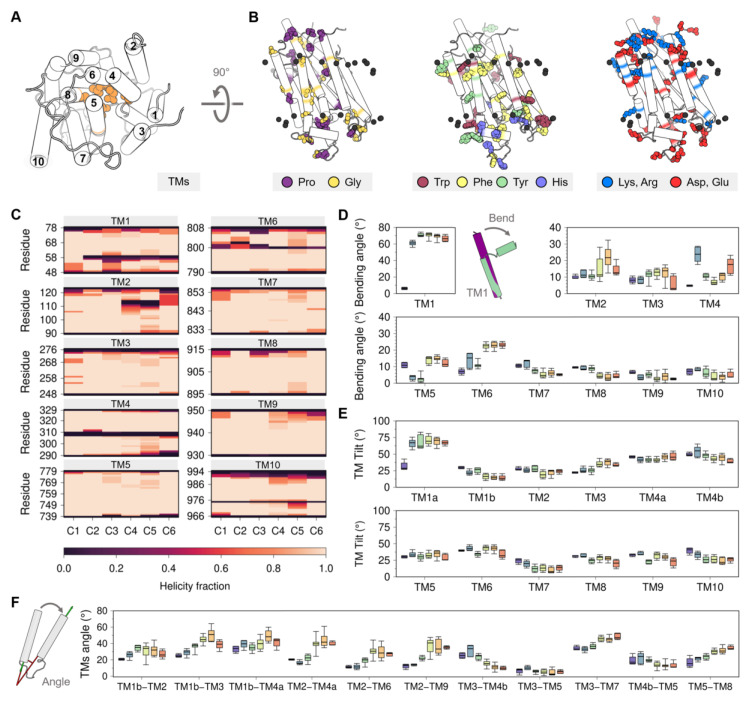
(**A**) Cytosolic view of the transmembrane domain with labeled TM helices. (**B**) Topological distribution of proline, glycine, aromatic, and charged residues. (**C**) Average helicity fraction of the TM helices in the six clusters. Distribution of the (**D**) bending and (**E**) TM tilt angles of the ten TM helices. (**F**) Angle between the helices of most representative TM helix pairs. Box and whisker plots are colored using the cluster analysis in Figure 2 as reference (*C1*, purple; *C2*, blue; *C3*, green; *C4*, yellow; *C5*, orange; *C6*, red).

The transmembrane domain of SERCA isoforms 1a and 2a is structurally characterized by ten TM helices (Figure 4A), while the SERCA2b isoform exhibits eleven TM helices [60,61,62]. The TM domain is divided into two main regions: (a) A mobile region formed by helices TM1–TM6, which plays a central role in controlling access to the Ca^2+^ binding sites and also contains the energy-transduction domain, a network of SERCA residues that is essential for transport/ATPase coupling [32,63]; and (b) a relatively rigid region that includes helices TM7 through TM10. This region of the protein has been hypothesized to serve as an anchor that provides stability to the pump during the catalytic cycle of the pump [22,64]. Lastly, helix TM11 of SERCA2b has been shown to specifically modulate the activity of the homologous SERCA1a; thus, it was conferred a regulatory role [65]. Nevertheless, the only crystal structure of SERCA2b has been reported to date (*5ztf*); this structure has the first ten TM helices and a small fragment of TM11 (residues F1018 to Y1030) [66], so the precise orientation of this helix remains unidentified. 

The amino acid composition and distribution of the TM domain of SERCA are typical for many transmembrane proteins [67] and include functionally important residues including proline, glycine, arginine, lysine, as well as aromatic residues (Figure 4B). Glycine and proline residues are commonly found in TM regions [68,69], which is surprising because these residues usually destabilize α-helices found in soluble proteins [70,71,72]. Nonetheless, the insertion of proline and glycine residues in the TM helices increases the plasticity of the membrane-bound domains and facilitates conformational transitions between functionally relevant states of the protein [73]. Proline residues are located at TM4 (P308 and P312), TM6 (P803), and TM10 (P976) of the SERCA structure; the loss of the main chain hydrogen bridge mediated by these residues leads to the formation of transmembrane kinks [74]. Similarly, prolines at the ends of TM9 (P926, P927, P952, and P954) are responsible for the helix disruption and the formation of small helical loops in the luminal and cytosolic regions. SERCA has 11 glycine residues in the TM region, of which three are located near the proline kinks (G310, G801, and G979), favoring the plasticity of the interrupted helix regions, and other three form a GXXGG motif in TM7 (G841, G844, G845) that permits the close interaction with TM5 (G770) [75]. At the membrane surface, SERCA contains interfacial phenylalanine, tyrosine, and tryptophan residues; these residues are important as they form an aromatic belt that guides anchoring, insertion, and orientation of the protein in the lipid bilayer [76,77]. In addition to these functional roles, aromatic residues found in the TM1-TM2 loop (Y29 and Y31) also participate in intermolecular interactions involving SERCA and its regulatory proteins [78]. Positively charged residues arginine and lysine at the protein-membrane interface play a key role in the structural stability of membrane-embedded helices. The TM domain of SERCA contains arginine and lysine on both cytosolic and luminal faces, with a higher density of these residues in the cytosolic region than in the luminal side. This distribution of positively charged residues corresponds to that proposed in the “positive-inside rule” [79,80], which postulates that positively charged residues preferentially populate the cytoplasmic side of TM helices. Both cytosolic and luminal sides of the TM region are rich in negatively charged aspartate and glutamate residues, as well as the TM core of the protein where the Ca^2+^ sites are located. There is consensus on the role of negatively charged residues for binding of Ca^2+^ ions from the cytosol and their subsequent translocation during active transport [21,81,82]. Yet, enrichment of acidic residues in the TM domain of SERCA is interesting because it has been proposed that a sufficiently large negative charge can overturn the positive-inside rule [79,80], so it is possible that negative residues are topologically active to a certain degree during the structural transitions of SERCA. 

Analysis of the crystal structures shows that the TM domain of SERCA undergoes subtle structural changes during the transitions between E1 and E2 states. These changes can be clearly visualized in the RMSD matrix of this region (Figure A2 of Appendix B), but we propose to use alternative metrics to identify state-specific changes in the TM domain, including helicity, bending angle, tilt angle, and interhelical angle of the TM helices. The use of these metrics may help to provide a more quantitative description of the structural changes in the TM domain that occur during the catalytic cycle of the pump. In this section, we provide a brief discussion of these metrics in relation to the clusters provided by the analysis of the crystal structures of the pump. 

#### 3.2.1. Changes in TM Helix Helicity and Bending Angle

Membrane proteins are able to undergo structural changes owing to the presence of intramembrane kinks and changes in the secondary structure of individual helices in the TM domain [83]. SERCA contains five TM helices with low helical segments in the intramembrane region caused by the conformational changes of the cytosolic A domain (TM1 and TM2) and proline kinks (TM4, TM6, and TM10) (Figure 4C). TM1 has a helix break at position 58, with a bending angle greater than 55.0° (Figure 4D), which leads to the formation of the Ca^2+^ entry pore in both catalytic states (*C2* to *C6* clusters) through the exposition of the negatively charged residues embedded in the TM domain. Bending of helix TM1 results in the formation of small (TM1a, residues 48 to 55) and large (TM1b, residues 61 to 78) TM segments. Rotation of the A domain during the transition from the E1 state (*C1* to *C3*) to E2 state (*C4* to *C6*) induces loss of helicity in the luminal amino acids R110–N114 and bending of helix TM2. TM3 does not show substantial changes in its helicity, but a slight increase in its bending occurs from *C2* to *C5* and finally a return to a more extended form in *C6*. However, the crystallographic data is in contrast with previous studies showing that helix TM3 is substantially more dynamic during the structural transitions of the pump. Specifically, experiments and coarse-grained simulations using insertion mutants in the A-TM3 linker region showed that TM3 plays a functional role in the E2-to-E1 transition through a mechanism that involves entropy–enthalpy compensation [84,85]. A more recent study showed that TM3 undergoes a change in the tilt angle that helps transmitting the protonation-dependent signal from the transport sites to the cytosolic headpiece required to initiate the E2-to-E1 transition of SERCA [57]. Changes in helicity of TM4 induces the formation of luminal (TM4a, aa 290 to 306) and cytosolic (TM4b, aa 311 to 329) helices. Although both TM4a and TM4b belong to the energy-transduction domain of the pump (i.e., the collection of SERCA residues that are essential for transport/ATPase coupling), TM4b has been hypothesized to play a central role in coupling/uncoupling of the pump [32,63]. The structural transitions experienced by SERCA during its catalytic function also involve bending of helix TM5, where an increase in the bending angle of this helix is observed during the transition of clusters *C2* and *C3* (E1 state) toward clusters *C4*–*C6* (E2 state). This observation is important because bending of helix TM5 has been suggested to be a signal transduction element required in the E2-to-E1 transition by disrupting the TM3–TM5 interface and modulating the dynamics of the A domain [57]. A substantially higher helical content is observed for TM6 cytosolic segment (residues G800–G808) in the E2 state structures when compared to E1 cluster groups. However, a complete loss of helicity at position D800 in the E2 state (clusters *C3*–*C6*) favors bending of TM6 by approximately 10°. Surprisingly, the proline kink of TM10 does not promote changes in its bending angle, although neighboring helix TM9 shows a small decrease in the bending angle upon transition of the pump toward the E2 state. Taken together, the helicity and bending angles of the TM helices capture key structural changes in the TM helices that may be helpful to establish changes in the TM domain during the catalytic cycle of SERCA.

#### 3.2.2. Changes in TM Tilt and Interhelical Angles

**Figure A4:**
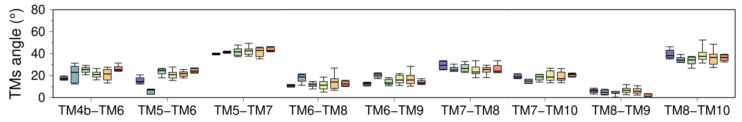
Interhelical angle of selected SERCA TM helix pairs.

Like intramembrane kinks, it has been shown that inclination of TM helices with respect to the membrane normal (tilt angle) or between TM helices (interhelical angle) are helpful metrics to determine changes in orientation and conformational states of membrane proteins [86,87]. In SERCA, tilt angles of the TM helices are similar in all structural clusters proposed here (*C1-C6*), so tilt angles do not represent a strong metric to identify structural changes in the TM domain associated with the structural transitions between the E2 and E1 states (Figure 4E). However, it is possible to determine concerted changes that occur during the transition between E1 and E2 states by analyzing the interhelical angle of TM helices (Figure 4F and Figure A4 of Appendix B). The most salient feature observed when using this metric is the roles of the luminal region of TM2 (residues 90 to 111) and TM4a, TM6, and TM9, where the transition between E1 and E2 states results in an increase of the interhelical angle between these TM helices (Figure 4F). This structural change induces separation of the luminal segments of helices TM2, TM4a, TM6, and TM9, as well as exposure of TM4a to the lipid bilayer and alterations to the topology of the regulatory protein binding site. Similarly, TM3 exhibits staggered changes in the interhelical angles with TM1b, TM4b, and TM7 that helps these helices come closer together during the transition from E1-2Ca (*C1)* to E2-P (*C6)*. This rearrangement, along with the increase in the angle between TM5 and TM8, also facilitates the formation of the inhibitory thapsigargin (TG)-binding site. Together, these observations indicate that changes in interhelical angles capture state-specific structural changes in the TM domain throughout the catalytic cycle of SERCA, and also serve as a proxy for identifying effector sites in the TM region of the pump. 

## 4. Interactions of SERCA with Ions, Small Molecules, and Regulatory Proteins

**Figure 5:**
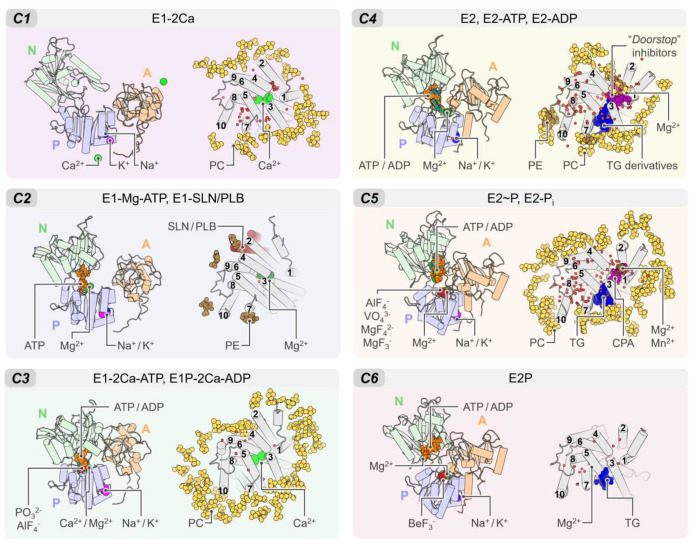
Summary of the binding sites located in the cytosolic and TM domains of SERCA. We show the cytosolic and transmembrane regions of SERCA bound to ions, lipids, small molecules, regulatory peptides, inorganic coordination entity, and water molecules in the six clusters described in this review. In all panels, the TM domain is shown from the cytosolic space in the membrane bilayer normal direction. Water molecules are shown as small red spheres.

**Figure 6:**
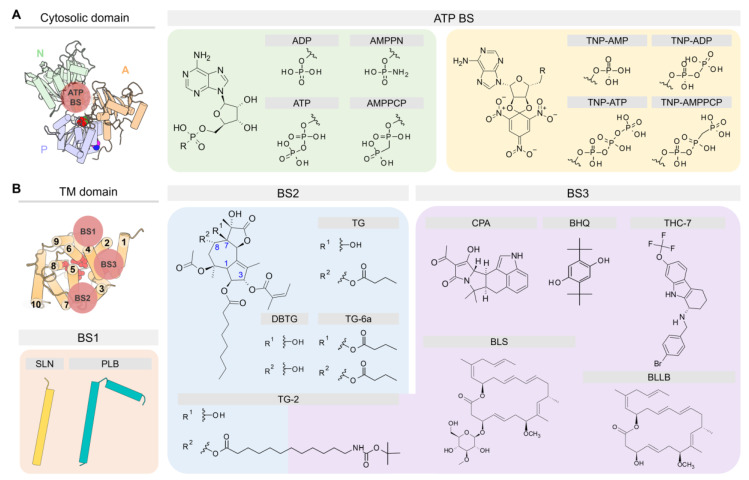
Chemical structures of small molecules cited in the text and schematic representation of their binding sites (BS) in the (**A**) cytosolic domain and (**B**) TM domain of SERCA.

In addition to the Ca^2+^ ions that bind to sites I and II, SERCA binds endogenous agents that are essential for the function and regulation, as well as molecules that modulate the activity of the pump (Figure 5). Most crystal structures of SERCA deposited in the PDB have been solved with different cations bound to well-defined regions of the pump, including the Ca^2+^ transport sites located in the TM domain (residues E309, E771, D800, and E908) which bind Ca^2+^, Mg^2+^, and H^+^ [18,28,88,89], the cytosolic P domain that features a K^+^-binding site (residues L711, K712, A714, and E732) that is required for dephosphorylation of the pump [90], and an Mg^2+^-binding site that participates in the reaction involving the use of ATP by SERCA [52,88,91]. Crystal structures of SERCA also feature non-native inorganic ions, including metal-fluoride and vanadate coordination molecules. These inorganic ions have been used to trap SERCA in intermediate states that are otherwise inaccessible in their native form, including: BeF_3_^−^ to obtain the structure of E2P in the ground state, AlF_4_^−^ and VO_3_^−^/VO_4_^3−^ to obtain the E2~P transition state, and MgF_3_^−^/MgF_4_^2−^ to solve the structure of the E2-P_i_ product state following SERCA phosphorylation at residue D351 [92,93]. Additional ions, such as Na^+^ and Mn^2+^, are also found in crystal structures of SERCA; however, these ions are often found in the buffers used to crystalize the protein and do not play a specific role in the function of the pump. In addition to inorganic ions, SERCA crystal structures have shown the presence of regulatory proteins PLB and SLN, nucleotide analogs, small-molecule effectors, water molecules, and organic additives (e.g., maltose). One of the most important accomplishments in the field is the ability to solve structures of SERCA bound to various lipids, including 1,2-dioleoyl-sn-glycero-3-phosphocholine (DOPC), 1-palmitoyl-2-oleoyl-sn-glycero-3-phosphoethanolamine (POPE), and n-decyl-β-D-maltoside (DM) bound in the TM domain. In nearly all the SERCA structures co-crystalized with lipids, only the phosphocholine (PC) and phosphoethanolamine (PE) headgroups were solved. While most crystal structures contain only a few lipids of the membrane, new advances in the field of SERCA crystallography now allow to identify the precise location of the lipids and the orientation of SERCA in the membrane throughout the catalytic cycle of the pump [94]. Based on the available crystallographic data, five specific lipid-binding sites have been identified in the SERCA TM domain: (1) A lipid-binding site formed by TM helices TM2, TM4, TM6, and TM9; (2) a site between TM1 and TM3; (3) a lipid site in helices TM7 and (4) TM10; and (5) a non-annular lipid-binding site located between the cytosolic regions of helices TM2 and TM4; this site has been observed only in the E2 state of SERCA. The interaction of these and other regions of the TM domain with lipids are mainly mediated by electrostatic interactions between arginine and lysine residues with phosphates, and by the aromatic belt of SERCA [94]. Due to the rich structural data acquired in these studies, it is now possible to identify several major binding sites (BS) located either at the cytosolic or TM domains of the pump (Figure 6). We briefly describe the location of these sites in the following subsections.

### 4.1. ATP Binding Site (ATP BS)

The cytosolic domain binds ATP in a cleft in the N domain formed by residues E442, F487, R489, K515, R560, and L562. In the crystal structures, two types of compounds have been used during the crystallographic experiments to mimic the cofactor binding in SERCA: (1) Adenosine monophosphate (AMP) derivatives and (2) trinitrophenyl (TNP) derivatives (Figure 6A). From the first group, [[5-(6-aminopurin-9-yl)-3,4-dihydroxyoxolan-2-yl]methoxy-hydroxyphosphoryl]oxyphosphonamidic acid (AMPPN) and the non-hydrolysable adenosine 5′-(β,γ-methylene)-triphosphate (AMPPCP) have been used to represent the ADP leaving group and the ATP binding molecule, respectively. In addition, the non-hydrolyzable ATP analog adenylyl imidodiphosphate (AMPPNP) has also been used in crystallographic studies of the pump [64]. Similarly, TNP derivatives contain AMP (TNP-AMP), ADP (TNP-ADP), ATP (TNP-ATP), and AMPPCP (TNP-AMPPCP) in their structure to resemble either nucleotide binding or its hydrolysis product. Except for the structural cluster *C1*, SERCA has been crystallized in the presence of nucleotide analogs. This finding has led to the conclusion that nucleotide is always bound to SERCA either in a catalytic or modulatory role [52]. The modulatory role has been supported by experiments showing that ATP-induced dynamics in the nucleotide-binding site decreases the energy barrier required for SERCA activation, modulating both Ca^2+^ binding (distal allostery) and SERCA phosphorylation (direct activation) [95,96]. 

### 4.2. Binding Sites in the TM Domain of SERCA

Small molecules and regulatory peptides that inhibit pump activity have been found predominantly in three sites: (1) Regulatory (canonical) binding site (BS1), (2) thapsigargin-binding site (BS2), and (3) “doorstop” inhibitors binding site (BS3) (Figure 6B). BS1 and BS2 sites are primarily hydrophobic grooves embedded in the membrane, whereas BS3 is a relatively small pocket located near the cytosolic side of the TM domain. Available crystal structures of the ligand-bound complexes show that regulatory proteins SLN and PLB occupy site BS1 in the E1 state (e.g., cluster *C2*), whereas small molecules are primarily bound to sites BS2 and BS3 in the E2 state of the pump (e.g., clusters *C4* to *C6*). We describe the location of these sites and their functional role in regulation and inhibition of SERCA.

#### 4.2.1. Regulatory Binding Site (BS1)

This site binds regulatory proteins SLN and PLB, and it is located between helices TM2, TM4, TM6, and TM9 of SERCA, resulting in inhibition of the activity of the pump. Crystallographic data by three independent groups revealed that SLN and PLB interact with SERCA primarily through hydrophobic contacts, although inhibition and regulation occur primarily at the cytosolic region of the complex and involve specific polar interactions between a highly conserved asparagine of the regulatory peptide (SLN-N11 and PLB-N34) and SERCA residues T805 and G801 [29]. Crystal structures have also shown that the C-terminal extension of SLN and PLB also interact with SERCA via polar interactions with the luminal region of the pump, either playing a stabilizing or a regulatory role [97,98,99,100]. The binding of these regulatory peptides was only identified in those SERCA structures clustered in *C2*; however, cluster-based analysis of crystal structures of SERCA showed that groups *C1*, *C3*, *C4*, and *C5* contain lipid molecules bound to this regulatory site (see Figure 5). This suggests that this site remains partially formed in both the E1 and E2 states of the pump, thus allowing regulatory proteins to stay bound to SERCA throughout the catalytic cycle. This mechanistic hypothesis has been supported by cross-linking experiments, ATPase activity, and Ca^2+^ transportation measurements, suggesting that SLN and PLB can bind to various biochemical states of SERCA with distinct effects on the pump’s kinetic properties [35,101,102].

An important difference observed in crystal structures of SERCA regulatory complexes is either the presence (e.g., SERCA–SLN, *4h1w* and *3w5a*) or absence (e.g., SERCA–PLB, *4kyt*) of Mg^2+^ ions bound to the transport sites of the pump. Based on these studies, it was proposed that these regulatory proteins regulate SERCA through different mechanisms: SLN inhibits SERCA by stabilizing an Mg^2+^-bound intermediate state [28,29], whereas PLB populates a metal ion-free state that is stabilized by protonation of acidic residues in the transport site [27]. However, molecular dynamics simulation and experimental studies have shown that an Mg^2+^-bound SERCA state does not represent a functional intermediate of the pump because (1) coordination at sites I and II is optimized for Ca^2+^ but not for Mg^2+^, (2) the coordination geometry and high desolvation cost for Mg^2+^ disfavor Mg^2+^ binding in the transport sites, (3) binding of Mg^2+^ to the transport sites most likely induces inhibition of the pump, and (4) based on a best-fit state model of SERCA turnover, it is likely that an Mg^2+^-bound state is not an intermediate in SERCA’s reaction cycle [13,103,104,105,106]. More recently, protein pKa calculations and microsecond-long MD simulations have shown that SLN and PLB stabilize a metal ion-free SERCA state protonated at residue E771 but ionized at E309 and D800 [107]. These observations had led to the conclusion that SERCA–PLB and SERCA–SLN populate a similar metal ion-free intermediate state at physiological conditions, and that this mechanism is utilized by both PLB and SLN to regulate SERCA in the cell. More recently, molecular simulations of the SERCA–PLB complex at high Ca^2+^ concentrations show that Ca^2+^ binds to transport site I, recruits E771 and D800, disrupts key inhibitory contacts involving the conserved PLB residue N34, and destabilizes the protonated, metal-ion free state of the complex [108]. This evidence has provided a dynamic structural model in which H^+^ and Ca^2+^ exchange at the transport sites, which is a switch that rapidly shifts the equilibrium between inhibited and noninhibited states of the SERCA–PLB complex.

#### 4.2.2. Thapsigargin Binding Site (BS2)

The TM domain of SERCA has a site that is highly specific for thapsigargin (TG), a sesquiterpene lactone isolated from the root of the plant *Thapsia garganica,* a potent non-competitive inhibitor of SERCA [109]. This inhibitor binds to a groove formed by TM helices TM3, TM4, TM5, and TM7, and TG is stabilized in this pocket through hydrophobic interaction with residues F256, L260, V263, Ile761, V769, I829, F834, and M838 [19,110]. Among these residues, F256 plays a critical role in binding TG to SERCA; for instance, mutagenesis studies have shown that SERCA mutant F256V induces significant reduction in the inhibitory activity of TG [111]. In addition to native TG, derivatives of this compound have been synthesized and co-crystallized with SERCA, thus providing additional insights into the binding modes and interactions of inhibitors in this site. Some of these chemical modifications include the hydrolysis of the butanoyl group at position 8 (DBTG) or the addition of a butyryloxy substituent at position 7 (TG-6a) [112,113]. Prior to these modifications, another group hydrolyzed the butanoyl group at position 8 of TG for its further acylation with 12-*tert*-butoxycarbonyl-aminododecanoic acid (TG-2) in order to reach the BS3 site [114].

Unlike regulatory peptides, TG and its derivatives have been observed in crystal structures of the E2 state of SERCA (i.e., clusters *C4* to *C6*). This observation has suggested that TG inhibits SERCA primarily in the E2 state, where it forms and stabilizes a catalytically inactive dead-end complex with the pump [115]. This structure-based hypothesis is consistent with live-cell experiments, showing that TG stabilizes an E2-like state that is incompatible with SERCA activation [58]. Nevertheless, these experiments have also shown that SERCA bound to the TG is able to populate two major structural states, whereas X-ray crystallography and electron microscopy have identified only a single structural state of the inhibited complex [58]. More recently, a computational study has suggested that TG inhibits SERCA via an allosteric mechanism in which TG induces changes in the structural dynamics of the N domain that prevents nucleotide binding to the pump [49]. These studies are complementary to crystallographic data and now provide a more accurate picture of the coupling between structural and biochemical states of TG-bound SERCA and suggest that the complex exists as a dynamic ensemble of diverse, yet catalytically inactive structures of the pump. 

#### 4.2.3. “Doorstop” Inhibitors Binding Site (BS3)

The so-called “doorstop” inhibitors are a group of small molecules that bind to the site located between TM1, TM2, TM3, and TM4 helices of SERCA [110]. This group of inhibitors prevents active site residues of SERCA from adopting an active conformation, thereby structurally blocking the function of the protein rather than working directly as competitive inhibitors binding to the active site. Similar to TG, the molecules inhibit SERCA by stabilizing an E2-like state (i.e., clusters *C4* and *C5*) and preventing TM1 from undergoing bending required for activation of the pump (see Figure 4) [116]. These “doorstop” inhibitors, which include the α-cyclopiazonic acid (CPA) and the 2,5-di-*tert*-butylhydroquinone (BHQ), have been widely used as reference compounds in experimental assays to understand the functional cycle of SERCA and the effect of its inhibition on calcium transport [117]. In addition, the coordination of divalent cations by CPA observed in crystral structures *3fps*, *3fgo*, *3fpb*, *4bew*, *4ycl*, and *5mpm* suggests that the inhibitor could interfere with binding of Ca^2+^ to the transport sites. Other inhibitors include the marine glycoside biselyngbyaside (BLS), a naturally occurring compound isolated from the cyanobacterium *Lyngbya* sp. [118], as well as its analog biselyngbyolide B (BLLB); both compounds are potent SERCA inhibitors and bind to BS3 with a unique binding mode. More recently, the tetrahydrocarbazole derivative THC-7 was tested for antifungal activity and co-crystalized in the BS3 site of SERCA [51], thus demonstrating the therapeutic potential of this site for the design of novel “doorstop” inhibitors.

## 5. Challenges and New Opportunities in the Field

### 5.1. Role of Lipids in Regulation of SERCA Activity

The emerging view that lipids allosterically modulate protein activity, and protein−ligand and protein−protein interactions of membrane proteins [119,120,121,122], demands efforts in the field to determine the location of lipid-binding sites and their functional role in a native-like environment. There is extensive evidence showing that SERCA activity is sensitive to changes in lipid bilayer thickness, where a gradual decrease or increase in the length of the lipid chains both affected SERCA function [123,124,125], and to lipid composition, where changes in the PE:PC ratio induces changes in SERCA’s *V_max_* and Ca^2+^ affinity [126,127]. These studies support the notion that the composition and characteristics of the lipid bilayer play a major role in SERCA function; however, investigating the location and function of lipid-binding sites in atomic-level detail have proven difficult using traditional approaches. To overcome this limitation, early studies in the field used complementary X-ray crystallography and atomistic molecular simulations to demonstrate for the first time that SERCA minimizes the hydrophobic/hydrophilic mismatch by inducing local deformation of the membrane and by adapting to the membrane via structural rearrangement of side chains and formation of TM helix tilts [112]. More recently, complementary small-angle X-ray scattering, and atomistic molecular dynamics simulations were used to resolve the entire first layer of phospholipids surrounding the TM helices of SERCA. These studies showed that the TM domain tilts throughout the reaction cycle primarily by a belt of tryptophan side chains, and through arginine side chains that “snorkel” [128,129] from within the bilayer to use phospholipids as anchors for conformational switching [94]. These studies have provided key structural information regarding the role of lipids in SERCA function; nonetheless, there are several open questions that need more consistent experimental results, including (1) the interplay between lipid binding and regulation of SERCA in a native membrane environment [130], (2) the specific vs. non-specific role of cholesterol on SERCA activity [126], and (3) the functional role of unique non-annular lipid sites found in the E2 state of the pump [126]. A more detailed review of lipid interactions with SERCA and other P-type ATPases was recently published by Hossain and Clarke [131].

### 5.2. Structures of Functionally Important and Novel Regulatory Complexes Remain Elusive

PLB phosphorylation regulates SERCA function by inducing an order-to-disorder structural transition of the cytosolic domain of PLB, destabilizing an inhibitory T state in favor of the disordered, non-inhibitory R state [132]. The structure of SERCA bound to the TM domain of PLB was solved recently [27], but the cytosolic domain in the phosphorylated R state is intrinsically disordered, so the high-resolution structure of phosphorylated PLB bound to SERCA has remained inaccessible to experiments. There has been speculation that SERCA has a well-defined pocket that houses the disordered cytosolic domain of phosphorylated PLB [130] and that this specific interaction results in relief of SERCA inhibition. Recent studies complement these findings and show that phosphorylation of PLB does not induce measurable changes in the interaction of the TM domain with SERCA [133], thus suggesting that control of SERCA regulation by PLB phosphorylation is primarily due to structural shifts and interactions in the cytosolic domains of SERCA and PLB. These mechanistic hypotheses have not yet been tested in atomic-level resolution but lay the foundation for new studies.

In addition to PLB and SLN, newly discovered micropeptide regulators have been reported in the literature, including transmembrane proteins dwarf open reading frame (DWORF) [134], myoregulin (MLN) [135], endoregulin (ELN), and another-regulin (ALN) [136]. Most of these proteins have been characterized as SERCA inhibitors, but there are studies showing that DWORF is an activator of the pump in both in vitro and in vivo models [134,137]. Except for the stoichiometry of the complexes between these micropeptides and SERCA [138], the structure–function relationships of these new regulators remain unknown largely because of the absence of experimental structures of these micropeptides or their complexes with SERCA. Addressing these gaps and challenges will yield a complete picture of the mechanisms for SERCA regulation and will also open new venues for the development and discovery of regulator-based therapies targeting SERCA [137,139].

### 5.3. Structural Basis of Functional Divergence among Homologous SERCA Regulators

Structural mechanisms behind differential regulation of SERCA by PLB and SLN is not well characterized, so this remains one of the greatest gaps in the field. Experiments and molecular simulation studies have shown that the N-terminal domain of SLN plays a primary role in SERCA uncoupling, while the TM domain is necessary for binding and stabilizing the regulatory complex, as well as for inhibiting Ca^2+^ affinity [22,23]. SLN mutagenesis experiments have also shown that the C-terminal domain is essential for SERCA inhibition [24], and membrane localization and retention [25]. More recently, studies using crystal structures as a starting point have proposed hypotheses for distinctive interactions that underlie the fundamental functional differences between SLN and PLB [63]. These achievements, inspired in part by the recent success in the field of SERCA crystallography [28,29], now provide the field with novel hypotheses, including: (1) C-terminus is an essential element required for dynamic control of SLN regulatory function, (2) PLB and SLN allosterically control SERCA using different communication pathways, (3) SLN, but not PLB, alters Ca^2+^ binding to SERCA by inducing unique changes in the energy-transduction domain of the pump. Testing these hypotheses will ultimately answer the most critical question: Why are PLB and SLN so similar, yet so different in their functional outcomes?

### 5.4. Translational SERCA Research Needs Structural and Mechanistic Understanding of Therapeutic Modulation

Abnormal SERCA activity and regulation are implicated in several conditions affecting human health, including heart failure [140,141,142,143,144,145,146,147,148], muscular dystrophy [149,150,151], diabetes [152,153], Alzheimer’s disease [154,155,156,157,158,159], and Parkinson’s disease [160,161,162]. A common theme in these disorders is the atypical intracellular Ca^2+^ overload that induces changes in Ca^2+^ handling, alterations in the mitochondrial swelling, oxidative stress, and ultimately necrosis. Reactivation of Ca^2+^ transport has been proposed as a therapeutic approach to treat these devastating diseases, and modulation of SERCA is amenable by small molecules [163,164], thus making SERCA an attractive target to treat these conditions. However, there are several major challenges preventing the translation of mechanistic discoveries into viable therapies that target SERCA, including: (1) No information of protein motions and allosteric communication pathways underlying therapeutic modulation of SERCA; (2) inadequate knowledge of the topology of effector sites on SERCA that are amenable for therapeutic modulation; and (3) limited availability of chemical probes of well-defined potency, selectivity, and cell permeability that can be used to systematically interrogate small-molecule SERCA activation. Addressing these challenges using a complementary approach that includes X-ray crystallography, spectroscopy, and simulations, in concert with cell-based assays and in vivo disease models, will enable translational research through paving the way for the design of effective products that specifically target SERCA in the cell. 

## 6. Methods 

### 6.1. Database Curation

A total of 74 SERCA structures were retrieved from the PDB [165]. Incomplete side chains were added with the *repair_model* module of WHAT IF server [166]. Missing loops and other structural segments of PDBs: *2o9j*, *2oa0*, *3ba6*, *3tlm*, *4h1w*, *4kyt*, *4y3u*, *4ycm*, *4ycn*, *5a3r*, and *5ncq* were modeled using MODELLER v9.22 [167,168] and Swiss-Model web server [169]. Given the high sequence identity between SERCA isoforms (ID > 80.0%) and to keep the same number of amino acids in each structure (residues 1-994), we built *Oryctolagus cuniculus* SERCA1a homology models (UniProt: P04191-2 [170]) using the crystal structures of SERCA2a (*5mpm* and *6hxb*) and SERCA2b (*5ztf* and *6jju*) isoforms. SERCA1a contains only 10 TMs, therefore the fragment of TM11 (residues 1018 to 1030) solved in *5ztf* was not considered in this analysis. The produced homology models were used to carry out all the comparative analyses described below (Table A2).

### 6.2. Clustering Analysis

Backbone RMSD matrix was computed with GROMACS 5.1 software [171] using the backbone of PDB: *2c9m* as reference. The matrix was subsequently used to generate the clusters employing the *linkage* method with a cut-off value of 0.23 nm.

### 6.3. Movement of the Cytosolic Headpiece

Due to its high conformational conservation, all SERCA structures were aligned to the Cα atoms of *2c9m* TM7 (residues 831–855), TM8 (residues 895–915), TM9 (residues 933–948), and TM10 (residues 966–994). Translation distances and rotation angles were computed for the three cytosolic domains using *2c9m* as reference with the *draw_rotation_axis.py* python script developed by Pablo Guardado Calvo for the PyMOL software (http://pymolwiki.org/index.php/RotationAxis). A domain (residues 1–40 and 115–241), P domain (residue 300–356 and 603–738), and N domain (residues 357–602) were isolated from the pre-aligned structures for the calculation. The distance between the center of geometry (COG) of the domains was computed using PyMOL.

### 6.4. Orientation of the TM Domain

TM domain helicity was defined using the *dssp* module [172] implemented in GROMACS 5.1. The bending angles were computed from the four residues comprising the bottom, middle, and top sections of the helix for each TM. Tilt angles were calculated using *helanal_trajectory* library of MDAnalysis software [173,174] with the SERCA structures oriented in a lipid bilayer template computed from the PPM web server [175]. Finally, the angle between the TM helices was computed with the *anglebetweenhelices.py* python script developed by Thomas Holder for the PyMOL software (https://pymolwiki.org/index.php/AngleBetweenHelices). Crystal structures *5zmw* and *5ncq* were not included in this analysis.

### 6.5. Visualization

Figures were generated with PyMOL v1.7 [176] and Gnuplot v5.2 [177].

## Abbreviations

A domain	Actuator domain
ADP	Adenosine diphosphate
AMPPCP	Adenosine 5′-(β,γ-methylene)-triphosphate
AMPPN	[[5-(6-Aminopurin-9-yl)-3,4-dihydroxyoxolan-2-yl]methoxy-hydroxyphosphoryl]oxyphosphonamidic acid
AMPPNP	Adenylyl imidodiphosphate
ATP	Adenosine triphosphate
BHQ	2,5-Di-tert-butylhydroquinone
BLLB	Biselyngbyolide B
BLS	Biselyngbyaside
BS	Binding site
CPA	α-Cyclopiazonic acid
DM	n-Decyl-β-D-maltoside
DOPC	1,2-Dioleoyl-sn-glycero-3-phosphocholine
ER	Endoplasmic reticulum
FRET	Fluorescence Resonance Energy Transfer
N domain	Nucleotide-binding domain
P domain	Phosphorylation domain
PDB	Protein Data Bank
PC	Phosphocholine
PE	Phosphoethanolamine
PLB	Phospholamban
POPE	1-Palmitoyl-2-oleoyl-sn-glycero-3-phosphoethanolamine
RMSD	Root-mean-square deviation
SERCA	Sarcoendoplasmic reticulum calcium ATPase
SLN	Sarcolipin
SR	Sarcoplasmic reticulum
TG	Thapsigargin
TM	Transmembrane
TNP	Trinitrophenyl

## Appendix A

**Table A1:** Published SERCA structures classified using RMSD-based clustering analysis.

Cluster	State	PDB ID	Res. (Å)	Ca^2+^ Sites	ATP BS	BS1	BS2	BS3	Phosphorylation Site	Lipids	Refs
C1	E1-2Ca	2c9m	3.00	Ca^2+^	-	-	-	-	-	-	[52]
C1	E1-2Ca	1su4	2.40	Ca^2+^	-	-	-	-	-	-	[89]
C1	E1-2Ca	3j7t	3.40	Ca^2+^	-	-	-	-	-	-	[43]
C1	E1-2Ca	5xa7	3.20	Ca^2+^	-	-	-	-	-	DOPC ^c^	[94]
C2	E1-Mg-ATP	3w5b	3.20	Mg^2+^	TNP-AMP/Mg^2+^	-	-	-	-	POPE ^c^	[28]
C2	E1-PLB	4kyt	2.83	-	-	PLB	-	-	-	-	[27]
C2	E1-PLB	4y3u	3.51	-	-	PLB	-	-	-	-	[27]
C2	E1-SLN	3w5a	3.01	Mg^2+^	TNP-AMP/Mg^2+^	SLN	-	-	-	POPE ^c^	[28]
C2	E1-SLN	4h1w	3.10	Mg^2+^	AMPPCP ^c^/Mg^2+^	SLN	-	-	-	-	[29]
C3	E1P-2Ca-ADP	3ba6	2.80	Ca^2+^	AMPPN	-	-	-	Asp-PO_3_^2−^/Ca^2+^	-	[64]
C3	E1~P-2Ca-ADP	5xa8	3.20	Ca^2+^	ADP/Mg^2+^	-	-	-	AlF_4_^−^/Mg^2+^	DOPC ^c^	[94]
C3	E1~P-2Ca-ADP	2zbd	2.40	Ca^2+^	ADP/Mg^2+^	-	-	-	AlF_4_^−^/Mg^2+^	DOPC	[178]
C3	E1~P-2Ca-ADP	1t5t	2.90	Ca^2+^	ADP/Mg^2+^	-	-	-	AlF_4_^−^/Mg^2+^	-	[179]
C3 ^b^	E1-2Ca-ATP	5ztf	3.45	Ca^2+^	AMPPCP/Mg^2+^	-	-	-	-	-	[66]
C3 ^b^	E1-2Ca-ATP	6jju	3.20	Ca^2+^	AMPPCP/Mg^2+^	-	-	-	-	-	[66]
C3	E1-2Ca-ATP	1t5s	2.60	Ca^2+^	AMPPCP/Mg^2+^	-	-	-	-	-	[179]
C3	E1-2Ca-ATP	1vfp	2.90	Ca^2+^	AMPPCP/Mg^2+^	-	-	-	-	-	[14]
C3	E1-2Ca-ATP	3tlm	2.95	Ca^2+^	AMPPCP/Mg^2+^	-	-	-	-	-	[180]
C3	E1-2Ca-ATP	3ar2	2.50	Ca^2+^	AMPPCP/Ca^2+^	-	-	-	-	DOPC ^c^	[181]
C3	E1-2Ca-ATP	3n8g	2.58	Ca^2+^	AMPPCP/Ca^2+^	-	-	-	-	-	[82]
C3	E1-2Ca-ATP	4xou	2.80	Ca^2+^	AMPPCP/Ca^2+^	-	-	-	-	-	[182]
C3	E1-2Ca-ATP	6hef	3.54	Ca^2+^	AMPPCP/Ca^2+^	-	-	-	-	DOPC	[183]
C3 ^a^	E1-2Ca-ATP	6hxb	4.00	Ca^2+^	AMPPCP/Ca^2+^	-	-	-	-	-	[56]
C3	E1-2Ca-ATP	6rb2	3.20	Ca^2+^	AMPPCP/Ca^2+^	-	-	-	-	-	[54]
C4	E2	2eau	2.80	-	-	-	-	CPA	-	POPE ^c^	[184]
C4	E2	3w5c	2.50	-	-	-	-	-	-	POPE ^c^	[28]
C4	E2	3w5d	2.45	-	-	-	-	-	-	POPE ^c^	[28]
C4	E2	4ycl	3.25	-	-	-	-	CPA/Mg^2+^	-	-	[184]
C4	E2	4ycm	3.20	-	-	-	-	BLS	-	POPE ^c^	[118]
C4	E2	4ycn	3.50	-	-	-	-	BLLB	-	POPE ^c^	[118]
C4	E2-TG	1iwo	3.10	-	-	-	TG	-	-	-	[19]
C4	E2-TG	2agv	2.40	-	-	-	TG	BHQ	-	POPE ^c^	[18]
C4	E2-TG	2c8l	3.10	-	-	-	TG	-	-	-	[52]
C4	E2-TG	2ear	3.10	-	-	-	TG	-	-	-	[184]
C4	E2-TG	2eat	2.90	-	-	-	TG	CPA	-	-	[184]
C4	E2-TG	2yfy	3.10	-	-	-	DBTG	-	-	-	[112]
C4	E2-TG	4j2t	3.20	-	-	-	TG-6a	-	-	POPE ^c^	[113]
C4	E2-TG	4uu0	2.50	-	-	-	TG	-	-	-	[185]
C4	E2-TG	5xab	3.20	-	-	-	TG	-	-	DOPC ^c^	[94]
C4	E2-TG	5zmv	3.30	-	-	-	TG	-	-	POPE ^c^	[50]
C4	E2-ADP-TG	3ar3	2.30	-	ADP/Mg^2+^	-	TG	-	-	POPE ^c^	[181]
C4	E2-ADP-TG	3ar5	2.20	-	TNP-AMP	-	TG	-	-	POPE ^c^	[181]
C4	E2-ADP-TG	3ar6	2.20	-	TNP-ADP/Mg^2+^	-	TG	-	-	POPE ^c^	[181]
C4	E2-ATP-TG	2c88	3.10	-	AMPPCP/Mg^2+^	-	TG	-	-	-	[52]
C4	E2-ATP-TG	2c8k	2.80	-	AMPPCP/Mg^2+^	-	TG	-	-	-	[52]
C4	E2-ATP-TG	2dqs	2.50	-	AMPPCP/Mg^2+^	-	TG	-	-	POPE ^c^	[186]
C4	E2-ATP-TG	3ar4	2.15	-	ATP/Mg^2+^	-	TG	-	-	POPE ^c^	[181]
C4	E2-ATP-TG	3ar7	2.15	-	TNP-ATP	-	TG	-	-	POPE ^c^	[181]
C4	E2-ATP-TG	4uu1	2.80	-	AMPPCP/Mg^2+^	-	TG	-	-	DOPC ^c^	[185]
C4	E2-ATP	2by4	3.30	-	AMPPCP/Mg^2+^	-	TG-2	TG-2	-	-	[114]
C4	E2-ADP	3fps	3.20	-	ADP/Mg^2+^	-	-	CPA/Mg^2+^	-	-	[187]
C4	E2-ADP	2oa0	3.40	-	ADP/Mg^2+^	-	-	CPA	-	-	[188]
C5	E2~P-TG	1xp5	3.00	-	-	-	TG	-	AlF_4_^−^/Mg^2+^	-	[20]
C5	E2~P-TG	5xa9	3.20	-	-	-	TG	-	AlF_4_^−^/Mg^2+^	DOPC ^c^	[94]
C5	E2~P-TG	5xaa	3.20	-	-	-	TG	-	AlF_4_^−^/Mg^2+^	DOPC ^c^	[94]
C5	E2~P-TG	2zbg	2.55	-	-	-	TG	-	AlF_4_^−^/Mg^2+^	-	[189]
C5	E2~P-TG	3n5k	2.20	-	-	-	TG	-	AlF_4_^−^/Mg^2+^	-	[82]
C5	E2~P-ATP	3b9r	3.00	-	AMPPCP	-	-	-	AlF_4_^−^/Mg^2+^	-	[64]
C5	E2~P-ADP-TG	3ar8	2.60	-	TNP-AMP	-	TG	-	AlF_4_^−^/Mg^2+^	-	[181]
C5	E2~P-ATP-TG	5a3q	3.05	-	TNP-AMPPCP/Mg^2+^	-	TG	-	VO_4_^3−^/Mg^2+^	-	[92]
C5	E2~P-ATP-TG	5a3s	3.30	-	TNP-ATP/Mg^2+^	-	TG	-	VO_4_^3−^/Mg^2+^	-	[92]
C5	E2-P_i_	2o9j	2.65	-	-	-	-	CPA	MgF_4_^2−^/Mg^2+^	-	[188]
C5 ^a^	E2-P_i_	5mpm	3.30	-	-	-	-	CPA/Mg^2+^	MgF_3_^−^/Mg^2+^	-	[56]
C5	E2-P_i_-ADP-TG	1wpg	2.30	-	ADP/Mg^2+^	-	TG	-	MgF_4_^2−^/Mg^2+^	-	[178]
C5	E2-P_i_-ATP	3fgo	2.50	-	AMPPCP	-	-	CPA/Mn^2+^	MgF_4_^2−^/Mg^2+^	-	[187]
C5	E2-P_i_-ATP	3fpb	2.55	-	ATP	-	-	CPA/Mg^2+^	MgF_4_^2−^/Mg^2+^	-	[187]
C5	E2-P_i_-ATP	4bew	2.50	-	AMPPCP	-	-	CPA/Mn^2+^	MgF_3_^−^/Mg^2+^	-	[190]
C6	E2P-TG	2zbf	2.40	-	-	-	TG	-	BeF_3_^−^/Mg^2+^	-	[189]
C6	E2P-ADP-TG	3ar9	2.60	-	TNP-AMP	-	TG	-	BeF_3_^−^/Mg^2+^	-	[181]
C6	E2P	2zbe	3.80	-	-	-	-	-	BeF_3_^−^/Mg^2+^	-	[189]
C6	E2P-Mg-ATP	5a3r	3.05	Mg^2+^	TNP-AMPPCP/Mg^2+^	-	-	-	BeF_3_^−^/Mg^2+^	-	[92]
C6	E2P-Mg	3b9b	2.65	Mg^2+^	-	-	-	-	BeF_3_^−^/Mg^2+^	-	[64]
NA	E2-TG	5zmw	2.50	-	-	-	TG	-	-	DM	[50]
NA	E2-ATP	5ncq	3.00	-	TNP-ATP	-	-	THC-7	-	DOPC	[51]
ND	E1-2Ca	4nab	3.50	Ca^2+^	-	-	-	-	-	DOPC	[44]
ND	E2	1kju	6.00	-	-	-	-	-	-	-	[42]

^a^ SERCA2a isoform, ^b^ SERCA2b isoform, ^c^ incomplete structure, NA: Not Assigned, ND: Not Determined.

**Table A2:** Root-mean-square distance (RMSD) of the Cα atoms between the SERCA1a models and the incomplete crystal structures.

PDB	RMSD (nm)	Cα Atoms	PDB	RMSD (nm)	Cα Atoms	PDB	RMSD (nm)	Cα Atoms
2o9j	0.01	939	4kyt	0.04	796	5mpm	0.01	866
2oa0	0.03	889	4y3u	0.05	830	5ncq	0.03	944
3ba6	0.02	873	4cym	0.02	903	5ztf	0.01	891
3tlm	0.02	877	4ycn	0.02	941	6hxb	0.01	920
4h1w	0.04	915	5a3r	0.02	889	6jju	0.09	850
